# ARAG, an Antioxidant-Rich Gel, Shows Superiority to Mepilex Ag in the Treatment of Deep Partial Thickness Burns without Sacrificing Antimicrobial Efficiency

**DOI:** 10.3390/antiox12061176

**Published:** 2023-05-30

**Authors:** Brian Michael Cartwright, Sean James Fox, Mary Jane Underdown, William Andrew Clark, Joseph Andrew Molnar

**Affiliations:** 1 ETSU Quillen College of Medicine, East Tennessee State University, Johnson City, TN 37614, USA; cartwrib@mail.etsu.edu; 2 Department of Rehabilitative Sciences, College of Clinical and Rehabilitative Health Sciences, East Tennessee State University, Johnson City, TN 37614, USA; 3 Department of Health Sciences, College of Public Health, East Tennessee State University, Johnson City, TN 37614, USA; foxsj@mail.etsu.edu; 4 Department of Plastic and Reconstructive Surgery, Wake Forest University School of Medicine, Winston-Salem, NC 27101, USA; 5 Wake Forest Institute for Regenerative Medicine, Wake Forest University School of Medicine, Winston-Salem, NC 27101, USA

**Keywords:** antioxidant, antimicrobial, burns, deep partial thickness burns, TPGS, wound healing

## Abstract

Current treatments for deep tissue burns are limited, and most serve only to enhance hydration or prevent bacterial growth. This leaves burn healing dependent on slow natural processes to debride the wound and reestablish the epidermal and dermal layers of the skin. Infections are well known to destabilize this process through a variety of mechanisms, most notably through increased inflammation and the resulting oxidative stress. In this study, we show that ARAG (an antioxidant-rich antimicrobial gel) can suppress the growth of multiple bacteria commonly found to infect burns (*Klebsiella pneumoniae*, *Proteus vulgaris*, *Pseudomonas aeruginosa*, and *Staphylococcus aureus*). This inhibition is comparable to that conferred by silver ion release from burn dressings such as Mepilex-Ag. We further show, using a porcine model for deep partial-thickness burns, that ARAG allows for enhanced wound healing over Mepilex-Ag, the current standard of care. Histological findings indicate this is likely due to increased wound debridement and dampening of late inflammatory processes, leading to more balanced physiologic healing. Taken together, these findings show promise for ARAG as a superior alternative to the current standard of care.

## 1. Introduction

Burns are one of the most common causes of trauma worldwide and account for a large number of intensive care unit admissions every year [1,2,3]. Of all burns, deep partial-thickness burns are some of the most serious and debilitating. They can have long-term physical, emotional, and psychological effects on patients. Existing treatments for deep partial-thickness burns, such as skin grafts, silver-containing dressings, and currently available topical medications, are often inadequate. Treatments, such as autologous skin grafts, require the generation of another wound elsewhere on the body, while other treatments, such as emollients and silver solutions, serve only to either keep the wound bed moisturized or to prevent bacterial growth [4,5]. Additionally, these treatments may not fully address the underlying damage to the skin, which can lead to further complications and decreased quality of life for patients [6,7,8].

One key factor in burn treatment is the prevention of infection, which is the greatest and most common source of increased morbidity and mortality for patients [9,10]. Current strategies to prevent infection include topical antibiotics, systemic antibiotics, and silver-eluting dressings; however, the bacteria that are routinely found to infect burn victims have been rapidly gaining resistance to both antibiotics and silver over time [11,12,13,14]. For this reason, there is a need for new treatment modalities to be developed that can circumvent these resistance mechanisms.

Another essential element in burn treatment is the prevention of a disproportionate inflammatory response. Burns are well known to induce high levels of inflammation [15,16,17]. While this is a natural bodily reaction and acts to prevent infection, it can have a detrimental impact on the healing process as well as cause severe systemic effects such as organ failure and death [15,16,17]. A good portion of the detrimental impact on wound healing is due to the production of reactive oxygen species, leading to a highly oxidative environment [15,18]. This can lead to additional tissue damage and further recruitment of inflammatory cells, resulting in a self-destructive inflammatory cycle [15,17,18].

Given these points, the ideal burn therapy would address not only the prevention of infection but also the counteracting of overactivation of the inflammatory response. To address this problem, we combined agents that have both antioxidant and antimicrobial properties into ARAG. ARAG contains several robust antioxidants (squalene, vitamin E, α-lipoic acid, and coenzyme Q_10_) as well as a nonionic surfactant, D-α-tocopheryl polyethylene glycol 1000 succinate (TPGS).

In this study, we investigate the antioxidant, healing, and direct antimicrobial properties of ARAG. To ensure clinical relevance to burn victims, we tested ARAG’s ability to inhibit the growth of bacteria commonly found to colonize burns: *K. pneumoniae*, *P. vulgaris*, *P. aeruginosa*, and *S. aureus* [11,12,19,20]. We then compared the efficiency of ARAG to ionized silver to test the in vitro efficacy of ARAG against a simulation of the current standard of care, Mepilex Ag. Overall healing augmentation was assessed in a porcine model for deep partial thickness burns, comparing ARAG directly to Mepilex Ag treatment. Lastly, histological investigations utilizing H&E and Masson’s trichrome staining of skin biopsies were performed to assess the inflammatory response and structural components within the wound. The findings of these studies, their potential clinical implications, and future directions will be discussed.

## 2. Materials and Methods

### 2.1. ARAG Formulation

ARAG was formulated to a final concentration of 9.5% deionized water, 8.5% squalene, 80.5% TPGS, and 1.5% lipophile (equal amounts α-lipoic acid, coenzyme Q_10_, and mixed tocopherols). TPGS was obtained from Antares Health, St. Charles, IL, USA. All other compounds were purchased through PureBulk in Rosburg, OR. TPGS was liquified through heating to 40 °C. Liquid components were then slowly mixed into the liquid TPGS using sterilized magnetic stir bars. Lipophile was then added to the resulting admixture and allowed to thoroughly dissolve before allowing the ARAG to congeal.

### 2.2. DPPH Measurement of Antioxidant Activity

The free radical scavenging ability of ARAG was determined by 2,2-diphenyl-1-(2,4,6-trinitrophenyl)-hydrazinyl (DPPH) (Cayman Chemical, MI, USA) quenching ability. A 0.1 mM stock solution of DPPH was made in methanol. 100, 200, 300, 400, 500, and 1000 µg/mL concentrations of ARAG and ascorbic acid (Sigma-Aldrich, St. Louis, MO, USA) were also prepared in methanol. 0.5 mL of ARAG or ascorbic acid solutions were added to 0.5 mL of a 0.1 mM DPPH solution and allowed to incubate at room temperature in the dark for 30 min. The absorbance of solutions was then measured at 515 nm. The scavenging activity was calculated based on the following: $[1-\left(Ab\;sample-Ab\;blank\right)/\left(Ab\;control\right)]$ × 100. Three replicates were performed for both ARAG and ascorbic acid.

### 2.3. Cell Culture, Viability Assays, and Western Blotting

BJ cells, primary human fibroblasts isolated from neonatal foreskin, were obtained from ATCC (CRL-2522) and maintained at 37 °C with 5% CO_2_ in Dulbecco’s modified Eagle’s medium (DMEM) supplemented with 10% fetal bovine serum and 1% penicillin/streptomycin. Hydrogen peroxide (30%) (Sigma-Aldrich, St. Louis, MO, USA) was freshly diluted in 1x phosphate buffered saline (PBS) before treatment of cells. ARAG and N-acetyl-L-cysteine (NAC) (Sigma-Aldrich, St. Louis, MO, USA) were dissolved to their target concentrations of 1 mg/mL ARAG and 5 mM NAC in complete cell culture media. Cells were pretreated with either ARAG or NAC for one hour before hydrogen peroxide treatment. 3-(4,5-dimethylthiazol-2-yl)-2,5-diphenyltetrazolium bromide (MTT) viability assays were performed at 12 h in triplicate following the manufacturer’s instructions (Invitrogen, cat#M6594). For western blot analysis, cells were harvested after a 12-hour treatment by scraping cells into media. The media was then centrifuged at 3000× *g* for 5 min at 4 °C. The media was removed, and cells were washed 3× in 1x PBS. Whole cell lysates were prepared by lysis of cells 1:5 in lysis buffer [50 mM Tris-HCl pH 8.0, 140 mM NaCl, 1% Triton X-100, 0.05% SDS, 1 mM ethylenediaminetetraacetic acid (EDTA), and 1× protease and phosphatase inhibitors] on ice for 10 min. Cell lysates were cleared by centrifugation at 10,000× *g* for 5 min at 4 °C. Fifty µg of protein was run on SDS-PAGE per lane for each treatment group and then transferred onto a polyvinylidene difluoride (PVDF) membrane for western blot analysis. Blots were routinely divided so that multiple proteins could be probed simultaneously. Antibodies to p27 (#3686), p53 (#2524), and actin (#4970) were obtained from Cell Signaling Technology. The antibody against cleaved-PARP1 (c-PARP1) (#552933) was obtained from BD Biosciences.

### 2.4. Planktonic Growth and Colony Formation Assays

*K. pneumoniae* (ATCC 13883), *P. vulgaris* (ATCC 25923), *P. aeruginosa* (ATCC 10145), and *S. aureus* (ATCC 13315) were all obtained from ATCC. Strains were revived by streaking bacteria on Luria broth (LB) agar, followed by incubation for 24 h at 37 °C. Liquid cultures of bacterial strains were achieved by inoculating 10 mL of LB media with a singular colony before allowing the bacteria to grow for 18–24 h at 37 °C with vigorous shaking (200 rpm). For planktonic growth curves, bacterial strains were inoculated 1:200 into 20 mL LB with or without 10% added ARAG. OD600 readings were taken at 0, 1, 2, 4, 8, and 12 h. Control cultures (mock inoculated LB) were plated on LB agar without dilution following a 12-hour incubation and showed no sign of growth after 24 h (data not shown). For colony formation unit (CFU) assays, bacterial strains were inoculated 1:100 into 10 mL of LB media without additive, with 10% ARAG, or with 50 ppm silver. Bacteria were grown for 24 h before being serially diluted and spread on LB agar plates. Plates were then grown for an additional 24 h at 37 °C before being counted. Control cultures (mock-inoculated LB) were plated without dilution following a 24-hour incubation and showed no sign of growth after 24 h. Four replicates were performed per treatment for both assays.

### 2.5. Deep Partial Thickness Burns

Four 25 kg Landrace/Yorkshire pigs obtained from North Carolina State University were subjected to standardized partial thickness burn injury using a heating block as previously described by Gaines et al. [21]. In brief, animals were sedated, provided analgesia, and maintained under anesthesia during the procedure. Using a controlled pressure device to hold the heating blocks, four standardized burns (3 to 4 cm in diameter) were created on each side of the animal. Wounds were treated with either ARAG, ARAG + micronized silver (1% *w*/*w* of 2–3.5 µM particles, Sigma-Aldrich), or Mepilex-Ag (Molnlycke, Norcross, GA, USA) immediately after burn creation. The wounds were dressed with non-adherent Telfa gauze and further wrapped with Kerlix gauze. To prevent unnecessary exposure, a protective jacket/saddle was placed over the backs of the animals to protect the burns for the duration of the study. The burns were debrided, and treatments were reapplied every three days for the duration of the study. Images of the burns were taken by digital photography at the start of the study and at the end of the study at 21 days. Elliptical biopsies containing both scars and unhealed burns were taken at days three and seven for histological analysis by routine H&E and Masson’s trichrome staining. Assessment of cellular proliferation was performed by Ki-67 (1:200, Cell Signaling, Cat#9449) labeling by immunohistochemistry according to 3,3′-diaminobenzidine (DAB) kit instructions (DAB-M, Millipore-Sigma). All pairs of wounds were stained and analyzed by light microscopy for inflammatory infiltration, wound debridement, and cellular proliferation. The images shown are representative of the trends seen while analyzing each treatment group. Humane care was provided to comply with the federal legislation (Animal Welfare Acts), the “Principles of Laboratory Animal Care” and the “Guide for Care and Use of Laboratory Animals”. This study was reviewed and approved by the Wake Forest University Institutional Animal Care and Use Committee (protocol and approval #A13-009—Anti-inflammatory Wound Dressing, approved 3 March 2014).

### 2.6. Statistical Analysis

All analyses for statistical analysis were performed in GraphPad Prism 9 (Dotmatics, San Diego, CA, USA). The assessment of statistical analysis in planktonic growth assays was determined by the *t*-test. For MTT and CFU assays, a one-way ANOVA with multiple comparisons was performed. Burns were assessed by a Wilcoxon matched-pairs signed rank test comparing wound and scar percentages obtained by calculating the area of the total wound subtracted from the remaining lesion to obtain scar percentages. One set of paired burns was omitted from the analysis due to inconsistency in burn depth. Significant *p*-values are listed in individual figures. The standard deviation is shown in all plots.

## 3. Results

### 3.1. ARAG Is a Potent Antioxidant Capable of Preventing Oxidative Damage-Induced Cell Death

**Figure 1:**
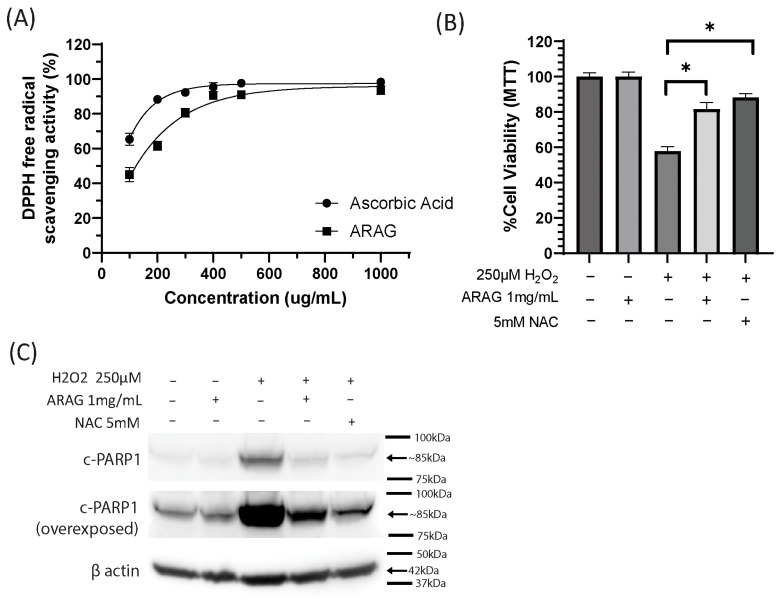
ARAG is a potent antioxidant capable of preventing oxidative damage induced cell death. (**A**) The antioxidant activity of ARAG was tested by a DPPH free radical scavenging assay. When compared to ascorbic acid, a known antioxidant, it was found that ARAG possessed good free radical scavenging ability. While this was lower than ascorbic acid at lower concentrations, it became near equivalent with an increased concentration of ARAG (98.3 ± 0.577% ascorbic acid vs. 93.6 ± 2.51% ARAG at 1 mg/mL). To test the ability of ARAG to prevent oxidative damage-induced loss of cell viability and cell death, BJ cells were treated with either 1 mg/mL ARAG or 5 mM NAC for one hour prior to a 12-hour treatment with 250 µM H_2_O_2_. (**B**) Results of MTT cell viability assays revealed that ARAG was highly effective at preventing hydrogen peroxide loss of viability and that this protective effect is near equivalent to that of the glutathione precursor NAC. *n* = 3, * *p* < 0.01 (**C**) Analysis of cell lysates by Western blot for the apoptotic marker c-PARP1 supported MTT findings showing a reduction in apoptosis in ARAG-treated cells exposed to hydrogen peroxide. This reduction was similar but less than that of NAC.

To determine the antioxidant potential of ARAG, we performed a DPPH free radical scavenging assay comparing ARAG to a known antioxidant, ascorbic acid (Figure 1A). At low concentrations (100, 200, and 300 µg/mL), ARAG possessed good neutralizing capabilities: 45.0 ± 4.00%, 61.7 ± 2.52%, and 80.7 ± 2.52, respectively. In comparison, ascorbic acid possessed higher neutralizing capabilities (65.3 ± 3.5%, 88.3 ± 1.53%, and 92.3 ± 1.53%) at the same concentrations. At 400 µg/mL and higher concentrations, ARAG quickly approached a plateau, showing only slightly lower free radical scavenging ability (93.7 ± 2.52%) than ascorbic acid (98.3 ± 0.578%) at 1 mg/mL.

To evaluate the protective effects of ARAG against oxidative damage, BJ cells were pretreated with either 1 mg/mL ARAG or 5 mM NAC for one hour before being subjected to a 12-hour treatment with 250 µM H_2_O_2_. (Figure 1B) MTT results showed that ARAG was able to prevent a good portion of the viability loss seen with H_2_O_2_ treatment. Additionally, this protective effect was similar to that of NAC, a known antioxidant and precursor to glutathione. (Figure 1C) Western blot analysis of cells similarly treated showed a large decrease in the amount of cleaved PARP1, a marker of apoptosis, in both the ARAG and NAC groups. This indicates that ARAG is protective against hydrogen peroxide-induced apoptosis but is not as efficient as high-dose NAC.

To further investigate the antioxidative potential of ARAG, we decided to assess its ability to inhibit cell death in the setting of marked oxidative stress. For this, normal human skin fibroblasts, or BJ cells, were utilized. The viability of cells was assessed by MTT assay 12 h post-hydrogen peroxide treatment (Figure 1B). It was found that ARAG was able to significantly reduce the loss of viability caused by hydrogen peroxide treatment. Furthermore, when compared to high-dose NAC, a precursor to the oxidative stress buffer glutathione, it was found that ARAG was only slightly less efficacious. In fact, this difference was in trend only, as no significant statistical difference was found between the ARAG and NAC groups. This was further confirmed by western blot analysis looking at cleaved PARP1 (c-PARP1), a protein marker of cellular stress and apoptosis (Figure 1C). In this assay, it was found that ARAG greatly reduced the level of c-PARP1 (lane 4) in comparison to the hydrogen peroxide-treated BJ cells (lane 3). This indicates that ARAG is an effective protectant against oxidative injury. When compared to NAC (lane 5), ARAG is seen to not have as protective an effect, which mirrors what was seen in the previous MTT assay. While this appears to be a large difference, this is only visually apparent in the overexposed image of c-PARP1, which was included to show baseline (lane 1) and that ARAG alone had no effect on cellular viability (lane 2).

### 3.2. ARAG Is Antimicrobial and Possesses Similar Efficacy to Ionized Silver

**Figure 2:**
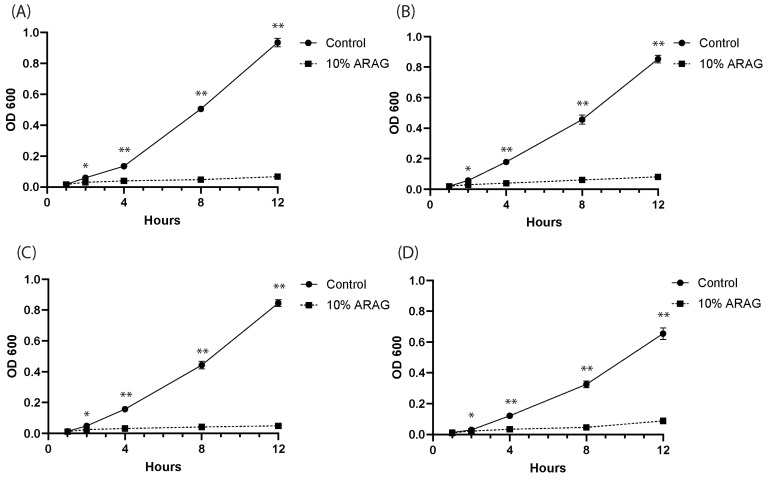
ARAG inhibits the planktonic growth of common burn bacteria. Overnight stock cultures of (**A**) *P. aeruginosa*, (**B**) *S. aureus*, (**C**) *K. pneumoniae*, and (**D**) *P. vulgaris* were inoculated into LB and grown at 37 °C with shaking for 12 h either in the presence or absence of 10% ARAG. OD600 measurements were taken at the time points indicated and normalized to the starting OD measurement (*n* = 4 individual cultures per group). It was found that 10% ARAG rapidly inhibited the growth of all four bacteria tested based on the culture density determined by OD600 measurement. Control cultures (mock inoculated LB) were plated on LB agar without dilution following 12 h of incubation and showed no sign of growth after 24 h (data not shown). * *p* < 0.01; ** *p* < 0.001.

To investigate the antimicrobial properties of ARAG, we first tested its ability to inhibit the planktonic growth of *K. pneumoniae*, *P. vulgaris*, *P. aeruginosa*, and *S. aureus* in liquid cultures (Figure 2). The addition of 10% ARAG resulted in a rapid and efficient inhibition of the growth of all four bacterial strains, as indicated by the difference in liquid culture absorbance at OD600 between treated and untreated groups. Significant inhibition in all trials was seen as early as 2 h into treatment and continued throughout the entire time course with increasing significance.

**Figure 3:**
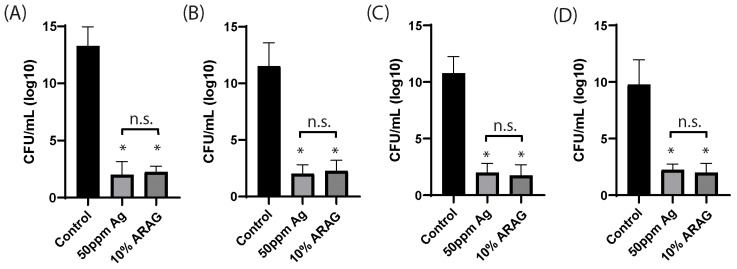
ARAG is non-inferior to ionized silver in inhibiting planktonic growth. Stock cultures of (**A**) *P. aeruginosa*, (**B**) *S. aureus*, (**C**) *K. pneumoniae*, and (**D**) *P. vulgaris* were normalized to 1 × 10^6^ and inoculated into 2 mL LB containing either saline, 50 ppm ionized silver, or 10% ARAG. Bacteria were then grown at 37 °C with shaking for 24 h before being diluted 10-fold and spread onto LB-agar plates. Plates were incubated for 24 h before being counted to determine CFU/mL. It was found that 10% ARAG significantly reduced the viability of all four bacteria tested, as indicated by the low number of CFUs. Ionized silver, the active anti-microbial component of Mepilex, showed similar results to the 10% ARAG group regarding comparison to the control; however, no significant difference was found between the 50 ppm ionized silver and 10% ARAG groups. Control cultures (mock-inoculated LB) were plated without dilution on LB agar following 24 h of incubation and showed no sign of growth after 24 h (data not shown). *n* = 4 individual cultures per group; * *p* < 0.05; n.s. = not significant; CFU = colony-forming units.

To further assess ARAG, we decided to test it against ionized silver for antimicrobial efficiency (Figure 3). Most clinically used silver-containing dressings elute silver ions to a sustained concentration of 70 ppm; however, at this concentration, silver is toxic not only to bacteria (30–40 ppm) but also to keratinocytes and fibroblasts (60 ppm) [22,23,24]. For this reason, we trialed an intermediate dosage of ionized silver (50 ppm) against 10% ARAG. Since silver interferes with OD600 readings, we chose to pursue colony formation assays as an alternative. It was found that both ARAG and 50 ppm Ag were able to significantly reduce the number of CFUs of all bacterial strains in comparison to the control. Interestingly, ARAG had similar CFU reducing properties at 50 ppm silver against all bacteria tested with silver, with ARAG affecting all strains to a similar degree. This indicates that ARAG is non-inferior to ionic silver and is efficient against bacteria regardless of their membrane properties.

### 3.3. ARAG Increases the Healing Rate of Deep Partial Thickness Burns

**Figure 4:**
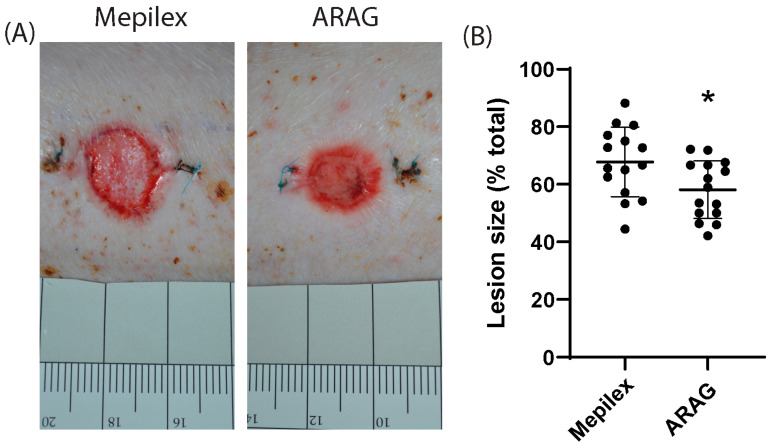
ARAG is superior to Mepilex for burn wound healing. (**A**) Representative images of burns at 21 days are shown. Wounds were assessed by digital photography, followed by quantification of area and comparison to the initial lesion size. (**B**) A comparison of paired burns showed a significant average reduction in lesion size of 9.65% in ARAG-treated burns over Mepilex-treated burns (*, *p* = 0.0031).

Using methods established by Gaines et al. [21], we conducted a non-inferiority trial for deep partial thickness burns in a porcine model comparing treatment with ARAG, ARAG + micronized silver, and Mepilex Ag. Given that the addition of silver to ARAG had no significant effect on healing rate (Supplemental Figure S1), a decision was made to pool the data for the ARAG and ARAG + micronized silver treatment groups. Using a pairwise comparison of parallel burns treated with either ARAG or Mepilex Ag, it was found that at 21 days, ARAG-treated burns showed a visual decrease in size when compared to Mepilex Ag samples (Figure 4A). When the remaining lesion was measured, it was found that there was a significant decrease in lesion size in ARAG-treated burns over Mepilex Ag-treated burns (Figure 4B).

### 3.4. ARAG Dampens the Late Inflammatory Response Leading to Organized Wound Debridement and Healing

To explore the process by which ARAG enhances wound healing, we decided to histologically examine paired biopsies taken at days three and seven from the wound edge. These elliptical biopsies, with sutures still visible in Figure 4A, were positioned so that both healed and unhealed portions could be investigated.

**Figure 5:**
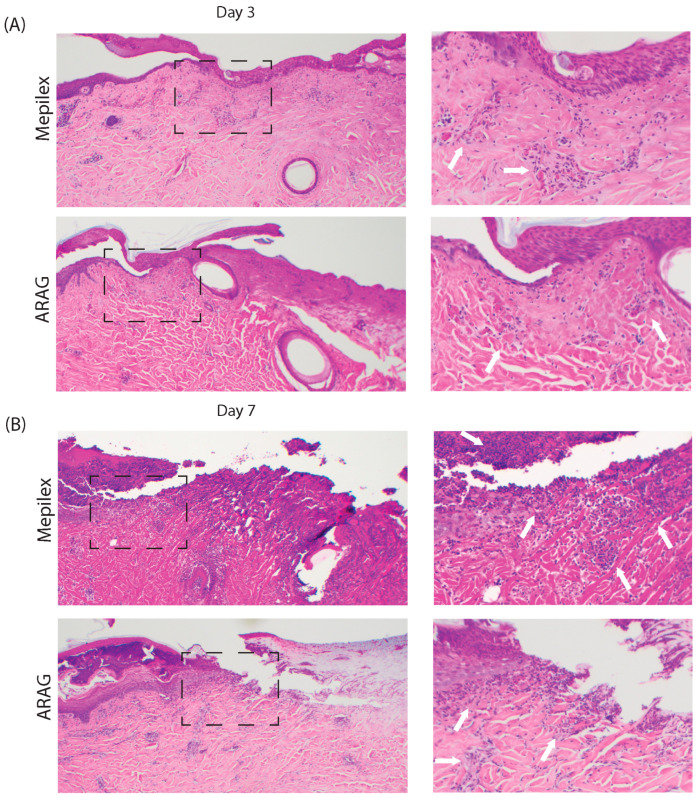
ARAG decreases the late inflammatory response. Representative images of paired skin biopsies stained with H&E at (**A**) day three and (**B**) day seven (4×). Zoomed images (10×) of the wound front (epidermal-dermal junction, black dashed box) are shown to the right. At day three, there is little difference between the levels of leukocytic infiltrate (hematoxylin, dark blue, stained cells) [white arrow] within the wound front. At day seven, there is a marked increase in leukocytic infiltration in both Mepilex and ARAG samples in comparison to day three; however, that infiltration is denser and present over a wider area within the tissue of Mepilex-treated animals.

Hematoxylin and eosin staining of biopsies at days three and seven was performed to analyze the basic histological evolution of the burn lesion (right) and adjacent healed skin (left) over time. Microscopic evaluation of the skin at day three (Figure 5A) showed a mild leukocytic infiltrate (hematoxylin, dark blue, stained cells) [white arrows] within the upper dermal layer of both Mepilex Ag- and ARAG-treated lesions; however, this infiltrate appears to be less in the ARAG-treated lesion. Interestingly, there is early denudation of the epidermis in the ARAG-treated group at day three. This is in stark contrast to the Mepilex Ag group, which still exhibits a near-full-thickness epidermis with a likely inflammatory infiltrate. With advancement to day seven (Figure 5B), the difference between groups becomes progressively more evident. Here we see that the entirety of the viewed dermis, both healed and active lesions, of Mepilex Ag-treated skin has a diffuse, full-thickness leukocytic infiltrate. This pattern of infiltration is not present in ARAG-treated lesions. Within these lesions, it appears that the leukocytic infiltration is diminished in both the healed and unhealed portions of the burn. Additionally, the infiltrate in ARAG-treated burns is focused primarily at the epidermal/dermal junction, where active healing is occurring. This more focused healing is further apparent in the regeneration of the epidermis with ARAG-treated burns, in which the newly healed skin possesses a normal reactive maturation pattern. This is not present in the late healing of the Mepilex Ag-treated burns, which appear parakeratotic under the remaining eschar.

**Figure 6:**
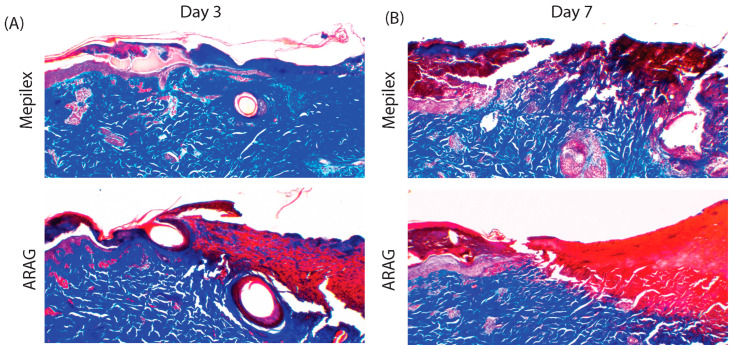
ARAG increases debridement at the burn healing front. Representative images (4×) of paired skin biopsies stained with Masson’s trichrome at (**A**) day three and (**B**) day seven. ARAG-treated burns display enhanced debridement and tissue remodeling, beginning at day three and increasing by day seven. This is demonstrated by the decreased or absence of collagen staining (blue) at the wound front (epidermal-dermal junction) at days three and seven, respectively. Unlike ARAG-treated samples, little to no collagen breakdown is seen at day three in the Mepilex group. At day seven, there is increased collagen breakdown within the Mepilex group; however, it is incomplete in comparison to ARAG.

Given the drastically different inflammatory patterns seen in Figure 5, we wanted to assess the debridement of the burns. Masson’s trichrome staining was performed on biopsy samples to investigate collagen (blue) staining as a surrogate marker for wound debridement and extracellular matrix remodeling. On day three (Figure 6A), it was found that Mepilex Ag-treated burns had little collagen breakdown within the lesion, whereas ARAG-treated samples possessed marked collagen breakdown, as shown by the replacement of blue staining with red staining. This collagen breakdown was even more prominent in ARAG-treated burns at day seven (Figure 6B), where no collagen staining is seen within the dermis directly adjacent to the advancing epidermal-dermal front. This is in stark contrast to the Mepilex Ag-treated lesions, where there is only partial breakdown of the dermis at the epidermal-dermal front with a large portion of collagen remaining.

### 3.5. ARAG Maintains Cellular Proliferation In Vivo and Decreases Markers of Oxidative Stress-Induced Senescence In Vitro

**Figure 7:**
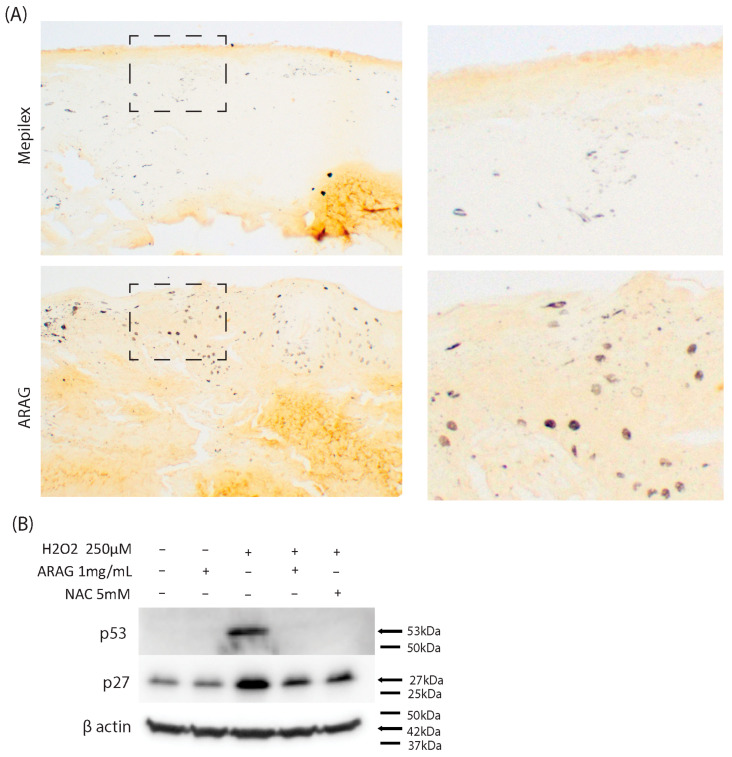
ARAG maintains cellular proliferation in vivo and decreases markers of oxidative stress-induced senescence in vitro. (**A**) Representative immunohistochemical staining (10×) of Ki-67, a nuclear marker for cell proliferation, of paired skin biopsies on day seven. Zoomed images of portions of the epidermal-dermal interface (black dashed box) are shown to the right. An increased number of Ki-67-labeled nuclei can be seen in the ARAG-treated group, indicating a higher rate of proliferating cells. To determine if this is due to the prevention of oxidative stress-induced cell cycle arrest and senescence, BJ cells were pretreated with either 1 mg/mL ARAG or 5 mM NAC for one hour before being subjected to a 12-hour treatment with 250 µM H_2_O_2_. (**B**) It was seen that ARAG and NAC both prevented the induction of p27 and p53 following hydrogen peroxide treatment. This suggests that blockage of oxidative stress by ARAG can prevent the upregulation of senescence-associated factors.

It is well known that aberrant and excessive inflammation induces cell cycle arrest, a senescence-associated secretory phenotype, and ultimately senescence itself [25,26,27,28]. To investigate the role of these factors in wound healing, we decided to gauge the difference in the cellular proliferation rate between Mepilex Ag- and ARAG-treated tissues (Figure 7A). Immunohistochemical staining of Ki-67, a nuclear marker of proliferating cells, revealed a higher number of proliferating cells within the ARAG-treated tissue. While this labeling does not specify which cell type is dividing, it implies a higher level of regeneration in the ARAG-treated samples. To determine if the antioxidant potential of ARAG is responsible for this, we investigated if ARAG could block the induction and upregulation of the senescence-associated markers p27 and p53 [26,28,29,30,31]. Both Western blot analyses (Figure 7B) revealed that ARAG was able to block the induction of p53 and upregulation of p27 caused by hydrogen peroxide. This was similar to NAC, demonstrating that prevention of this upregulation was antioxidant dependent.

## 4. Discussion

Treatment of deep partial thickness burns is difficult due to the risk of infection, prolonged inflammatory response, dehydration, and complete reliance on reepithelization from the edge of the wound [15,18]. Current treatments for deep tissue burns frequently only address one of these major concerns. Our study, however, has shown ARAG to be efficient at addressing all these concerns.

The antioxidant and anti-inflammatory components of ARAG are made up of squalene, vitamin E, α-lipoic acid, and coenzyme Q_10_. These are all compounds that have been shown to combat oxidative stress and excessive inflammation and promote more efficient wound healing [32,33,34,35,36,37,38]. When we compared the antioxidant capacity of ARAG to that of ascorbic acid, we found that ARAG behaves as a potent antioxidant capable of quenching free radicals. When compared to ascorbic acid, it is seen that at lower concentrations, ARAG has slightly less antioxidant potential; however, with increasing concentrations of ARAG, this gap in radical scavenging potential rapidly disappears. To test for the biological relevance of this scavenging potential, we investigated ARAG’s antioxidant capacity in a biological system by employing BJ cells, primary human skin fibroblasts. Results of both MTT and western assays (Figure 1B,C) showed robust protection of cellular viability and decreased cell death in comparison to the known antioxidant, NAC. Taken together, these results indicate that ARAG is a potent antioxidant capable of preventing oxidative stress-induced loss of viability and death.

ARAG was further found to have robust antimicrobial properties in vitro against all the common burn pathogens tested (Figure 2 and Figure 3). Given these results against Gram-positive and Gram-negative bacteria within this study, it is likely that ARAG would be effective against a wide array of other pathogens that were not tested. Of note, however, we did not test strains known to be resistant to silver compounds or antibiotics due to biosafety concerns. We hypothesize, however, that ARAG would have a similar effect on these strains, as the antimicrobial activity is likely due to the surfactant and/or emulsifying properties of the TPGS and other compounds within ARAG. Prior studies with similar compounds have shown that surfactants and emulsifying agents are highly capable of disrupting bacterial cell membranes, leading to either bacteriostatic or bactericidal effects [39,40,41]. For instance, many common hand soaps rely on the surfactant effects of fatty acids for their antibacterial properties [42]. Surfactant effects are also attributed to the antimicrobial activity of quaternary ammonium compounds, which have been gaining popularity due to their broad activity spectrum [43]. Emulsifying agents, such as chitosan/lethicin and sodium lauryl sulfate, have also been shown to have antimicrobial activity against an array of bacteria [44,45]. ARAG contains agents from both classes, making it highly likely that ARAG exerts its antimicrobial effect through similar mechanisms. While no antibiotics were used in this study, TPGS has also been used in a variety of antibiotic drug formulations where it has been found to greatly increase the efficacy of the antibiotics tested [46,47]. Therefore, it is possible that antibiotics could be compounded into ARAG if additional antibacterial, antifungal, or antiviral coverage was necessary.

When we tested the effectiveness of ARAG on wound healing, we found that ARAG is not only non-inferior to Mepilex Ag but superior to it regarding wound healing rate (Figure 4). To our surprise, there was a considerable difference in inflammatory cell infiltration between the two treatments, with fewer infiltrating cells found in the ARAG-treated burns (Figure 5). These findings imply that ARAG can modify the immune response. In doing so, fewer immune cells are recruited, and the ones that are recruited appear to be vastly more competent at inducing collagen breakdown and extracellular remodeling (Figure 6). Furthermore, this remodeling is limited only to the unhealed area and does not extend into the healed area, where it could cause detriment. This finding is interesting because the levels of collagen breakdown and debridement typically correlate with increased levels of inflammation and inflammatory infiltrate [15,18]; however, this was not observed. Our results instead show that ARAG-treated wounds, with less inflammatory infiltration than Mepilex-treated wounds, have a higher degree of collagen breakdown and wound debridement. This is important as proper debridement and wound bed maintenance are essential for ensuring appropriate reepithelization and future tissue integrity [15,18,48,49].

One possibility is that ARAG is influencing the immunological response within the burn wound to promote a more physiological and orderly immune response. In further support of this, components of ARAG have individually been found to affect various factors involved in wound healing. For example, squalene has been found to modulate pro- and anti-inflammatory processes to induce a more balanced activation of leukocytes within cutaneous lesions [33,34]. Additionally, vitamin E has been shown to mitigate the early hyper-inflammatory response to cutaneous wounds, thereby preventing the self-destructive inflammatory cycle known to induce further tissue damage and deter wound healing [15,17,18,37].

Another explanation for the increased healing rate seen in ARAG-treated wounds is that the direct antioxidant effect of ARAG is serving to prevent further tissue damage and dampen the oxidative environment within the healing tissue. Oxidative stress is well known to induce cell cycle arrest and upregulation of senescence-associated markers [29,31,32,33,34]. Our in vivo (Figure 7A) and in vitro (Figure 7B) studies show that tissue cell cycle activity is highly preserved in ARAG-treated wounds versus Mepilex-treated wounds and that ARAG lessens the upregulation of senescence-associated markers following hydrogen peroxide treatment, respectively. These results indicate that ARAG reduces oxidative stress and consequently decreases the pressure for cell cycle arrest and senescence, typically resulting from oxidative stress driven inflammation.

When consideration is given to these results, ARAG appears to be a perfect candidate for burn treatment: (1) it is directly antimicrobial against a variety of common burn pathogens; (2) it increases wound healing over the current standard of care; (3) it dampens the oxidative and inflammatory responses within the wound; and (4) it supports the maintenance of dividing cell populations, allowing for faster wound reepithelization.

## 5. Conclusions

ARAG is a potent, non-toxic antimicrobial gel capable of reducing oxidative stress, preserving cell viability, and increasing wound healing. While further studies are necessary to determine the exact molecular underpinnings involved in ARAG’s modulation of the inflammatory response and its effects on healing, our current findings show promise for the use of ARAG over the current standard of care. This is important not only from a healing aspect but also from a practical one. For example, the components used to make ARAG are relatively inexpensive in comparison to Mepilex Ag or silver sulfadiazine. This makes ARAG a far more economical approach to burn treatment, a concern that is constantly at the forefront given the rising costs of healthcare. Additionally, ARAG has a lower potential for side effects in comparison to current treatments as it is primarily composed of natural compounds. The same cannot be said of Mepilex Ag or silver sulfadiazine, both of which can cause skin discoloration, allergic reactions, and, in rare cases, anemia or leukopenia [50,51,52].

Future studies of ARAG will be directed at determining its immunomodulatory properties, molecular mechanism, and translational studies to assess ARAG’s potential in a clinical setting. In addition, we hope to further optimize our formulations to enhance the efficacy of ARAG for not only the treatment of burns but also for other wound categories.

## 6. Patents

The patent for the compound tested in this study is held by William A. Clark. US Patent Number 10,912,759 was awarded on 9 February 2021. “Topical Gel Compositions for the Treatment of Staphylococcus Infections”. Lavengel^®^ is a registered trademark with the United States Patent Office.

## Data Availability

The data presented in this study are available on request from the corresponding author.

## Supplementary Materials

The following supporting information can be downloaded at: https://www.mdpi.com/article/10.3390/antiox12061176/s1. Figure S1: The addition of micronized-Ag to ARAG does not significantly influence wound healing.
