# Supplementary material for: ARAG, an Antioxidant-Rich Gel, Shows Superiority to Mepilex Ag in the Treatment of Deep Partial Thickness Burns without Sacrificing Antimicrobial Efficiency
Source: Antioxidants (Basel). 2023 May 30;12(6):1176. doi: 10.3390/antiox12061176 (PMC10295424; doi:10.3390/antiox12061176)
Supplement: Supplementary file 1 [file antioxidants-12-01176-s001.zip › antioxidants-2374982-supplementary.pdf]

Supplementary Materials

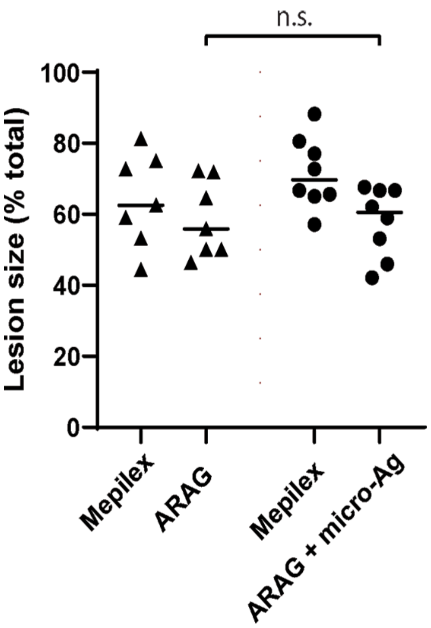

Supplementary Figure S1: Addition of micronized-Ag to ARAG does not significantly influence wound healing.

Burn studies of two pigs were conducted with ARAG alone while the other two pigs were conducted with ARAG + micronized-silver (micro-Ag). Paired comparison of ARAG (n=7) to ARAG + micronized-silver (n=8) shows that the two groups are not significantly different from each other by ANOVA with independent variable analysis. This supports the pooling analysis that is performed in **Figure 3**. n.s. = not significant
